# A Proof-Of-Principle Study of Epigenetic Therapy Added to Neoadjuvant Doxorubicin Cyclophosphamide for Locally Advanced Breast Cancer

**DOI:** 10.1371/journal.pone.0000098

**Published:** 2006-12-20

**Authors:** Claudia Arce, Carlos Pérez-Plasencia, Aurora González-Fierro, Erick de la Cruz-Hernández, Alma Revilla-Vázquez, Alma Chávez-Blanco, Catalina Trejo-Becerril, Enrique Pérez-Cárdenas, Lucia Taja-Chayeb, Enrique Bargallo, Patricia Villarreal, Teresa Ramírez, Teresa Vela, Myrna Candelaria, Maria F. Camargo, Elizabeth Robles, Alfonso Dueñas-González

**Affiliations:** 1 Unidad de Investigación Biomédica en Cáncer, Instituto de Investigaciones Biomédicas, Universidad Nacional Autonóma de Mexico UNAM, Instituto Nacional de Cancerologa INCAN, Mexico City, Mexico; 2 Division de Investigación Clinica, Instituto Nacional de Cancerología INCAN, Mexico City, Mexico; 3 Laboratorio de Desarrollo de Metodos Analiticos, FES-Cuautitlán, Universidad Nacional Autonóma de Mexico UNAM, CuautitlnIzcalli, Estado de México, Mexico; 4 Departamento de Tumores Mamarios, Instituto Nacional de Cancerología INCAN, Mexico City, Mexico; 5 Departamento de Patología, Instituto Nacional de Cancerología INCAN, Mexico City, Mexico; Ordway Research Institute, Inc., United States of America

## Abstract

**Background:**

Aberrant DNA methylation and histone deacetylation participate in cancer development and progression; hence, their reversal by inhibitors of DNA methylation and histone deacetylases (HDACs) is at present undergoing clinical testing in cancer therapy. As epigenetic alterations are common to breast cancer, in this proof-of-concept study demethylating hydralazine, plus the HDAC inhibitor magnesium valproate, were added to neoadjuvant doxorubicin and cyclophosphamide in locally advanced breast cancer to assess their safety and biological efficacy.

**Methodology:**

This was a single-arm interventional trial on breast cancer patients (ClinicalTrials.gov Identifier: NCT00395655). After signing informed consent, patients were typed for acetylator phenotype and then treated with hydralazine at 182 mg for rapid-, or 83 mg for slow-acetylators, and magnesium valproate at 30 mg/kg, starting from day –7 until chemotherapy ended, the latter consisting of four cycles of doxorubicin 60 mg/m^2^ and cyclophosphamide 600 mg/m^2^ every 21 days. Core-needle biopsies were taken from primary breast tumors at diagnosis and at day 8 of treatment with hydralazine and valproate.

**Main Findings:**

16 patients were included and received treatment as planned. All were evaluated for clinical response and toxicity and 15 for pathological response. Treatment was well-tolerated. The most common toxicity was drowsiness grades 1–2. Five (31%) patients had clinical CR and eight (50%) PR for an ORR of 81%. No patient progressed. One of 15 operated patients (6.6%) had pathological CR and 70% had residual disease <3 cm. There was a statistically significant decrease in global 5^m^C content and HDAC activity. Hydralazine and magnesium valproate up- and down-regulated at least 3-fold, 1,091 and 89 genes, respectively.

**Conclusions:**

Hydralazine and magnesium valproate produce DNA demethylation, HDAC inhibition, and gene reactivation in primary tumors. Doxorubicin and cyclophosphamide treatment is safe, well-tolerated, and appears to increase the efficacy of chemotherapy. A randomized phase III study is ongoing to support the efficacy of so-called epigenetic or transcriptional cancer therapy.

## Introduction

Aberrant gene transcriptions resulting from epigenetic changes are frequent events in the molecular pathogenesis of malignant transformation. DNA hypermethylation and histone deacetylation are critical for determining a closed chromatin structure responsible for or related with aberrant gene transcription in malignancies [1]. In counterpoint to genetic defects, the reversible nature of epigenetic aberrations constitutes an attractive therapeutic target; hence, a number of DNA methylation and histone deacetylase (HDAC) inhibitors are undergoing preclinical testing in combination for cancer therapy because it is clear that these drugs exert a synergistic effect on gene expression [2], [3] and tumor growth [4]–[8]. To date, only a limited number of clinical trials has been reported using both a demethylating and an HDAC inhibitor for treatment of hematological [9] and solid tumors [10]. Interestingly, preclinical reports have underscored that DNA methylation and HDAC inhibitors also possess the ability to potentiate radiation and chemotherapy [11]–[15]. In addition, it is known that chemotherapy resistance―either innate or acquired―requires expression changes in a large number of genes for its development; therefore, it has been hypothesized that epigenetic-mediated changes could be the responsible driving force for chemotherapy resistance; thus, epigenetic therapy has the potential to revert chemotherapy resistance [16].

In breast cancer, there is substantial evidence demonstrating the importance of epigenetic mechanisms in transcriptional regulation of critical tumor suppressor and growth regulatory genes. These genes include those that play crucial roles in DNA repair, cell-cycle regulation, cell growth, and cell-cell adhesion, which can all contribute to breast cancer tumorigenesis, metastasis, and resistance to therapy [17]–[20]. Thus, clinical testing of a combination of epigenetic agents together with classical cytotoxic chemotherapy in breast cancer appears desirable.

Primary systemic, neoadjuvant, or preoperative therapy is the standard of care in patients with operable and locally advanced breast cancer [21]. Comparable outcomes have been found for pre- and post-operative administration of various cytotoxic regimens [22], [23]; however, pre-operative therapy has the advantage of the assessment of pathological tumor response, which has been correlated with improved survival [24], [25]. Additionally, it provides an opportunity to evaluate biological markers after treatment. Therefore, in this proof-of-principle study we intended to evaluate the safety and biological and clinical efficacy of the epigenetic agents hydralazine and magnesium valproate plus neoadjuvant doxorubicin and cyclophosphamide in locally advanced breast cancer.

## Methods

### Patients

Eligible patients were 18 years of age and older, with histologically proven invasive T2-3, N0-2, and M0 (stages IIB–IIIA) breast carcinoma. Additional eligibility requirements included Eastern Cooperative Oncology Group performance status ≤2, absolute leukocyte count ≥4,000/mm^3^, platelets ≥100,000/mm^3^, hemoglobin ≥9.0 g/dL, total bilirubin, aspartate amino transferase and alanine amino transferase ≤1.5 the upper normal limit, creatinine ≤1.2 mg/dL or a calculated creatinine clearance of ≥60 mL/min, and written informed consent.

Patients were excluded if they referred a history of allergy to sulfas, hydralazine, or magnesium valproate, past or present condition of rheumatic disease, central nervous system disease, heart failure from aortic stenosis and postural hypotension as diagnosed by a physician, previous use of the experimental drugs, as well as if patients were pregnant or breast-feeding. Other exclusion criteria included uncontrolled systemic disease or infection. The protocol was approved by the Institutional Review Boards of the Instituto Nacional de Cancerologia Mexico and carried out in accordance with the Declaration of Helsinki, good clinical practices, and local ethical and legal requirements.

### Treatment plan

Once informed consent was signed, patients received a single oral 500-mg dose (two 250-mg tablets) of sulfamethazine early in the morning, and urine was collected within the ensuing 6 h for acetylator-status phenotyping, which was carried out as reported [26]. Afterward, patients began treatment (day –7) with a daily dose of a slow-release formulation of hydralazine tablets containing either 182 mg for rapid-acetylators or 83 mg for slow-acetylators. Magnesium valproate tablets of 700 mg were also administered as a slow-release formulation at a dose of 30 mg/Kb t.i.d. Both hydralazine and magnesium valproate were administered from day –7 until the last day of the fourth chemotherapy cycle.

### Chemotherapy

Chemotherapy consisted of four cycles of doxorubicin 60 mg/m^2^ and cyclophosphamide 600 mg/m^2^ at day 1 every 21 days. Antiemetic premedication was intravenous (i.v.) dexamethasone 16 mg and ondansetron 16 mg.

### Therapy duration and dose modifications

Patients received hydralazine and magnesium valproate until the last chemotherapy cycle, and were discontinued if the patient was unable to tolerate these despite dose modification, if the patient developed concurrent illness, or if the patient underwent changes in medical condition rendering them unacceptable for further treatment. Chemotherapy administration was delayed for 1 or 2 weeks in the event of any grade 3 or 4 hematological and non-hematological toxicity. In all these cases, however, hydralazine and magnesium valproate were continued. Only the dose of magnesium valproate was reduced 25% for grade 3 drowsiness, and was continued at the reduced level throughout the ensuing chemotherapy treatment. Routine or prophylactic use of colony stimulating factors was not allowed during treatment. Nonetheless, growth factor support was permitted to treat a neutropenic event. If the disease progressed during pre-operative therapy, chemotherapy was discontinued and patients underwent immediate surgery or radiation therapy. After final clinical-response assessment, patients were required to be operated on between 14 and 28 days after the last chemotherapy application. Thereafter, all patients received standard radiotherapy of the remaining breast tissue or to the chest wall. Adjuvant weekly paclitaxel 80 mg/m^2^ was administered for 12 weeks in patients with positive nodes after surgery. HER2-positive patients also received adjuvant trastuzumab. Tamoxifen 20 mg/day was administered in estrogen receptor-positive patients.

### Assessment

Baseline work-up included a complete history and physical examination, bilateral mammography and sonography of the breast, chest x-ray, liver ultrasound (US), and bone scintigraphy. Histologically, the tumor was evaluated with Scarff-Bloom-Richardson grading; HER2 and hormone receptors were assayed by immunohistochemistry. Laboratory assessment consisted of complete blood cell count and blood chemistry. Tumor assessment was performed within 2 weeks prior to initiation of transcriptional therapy. Clinical evaluation of palpable tumor and lymph nodes was repeated before each cycle and before surgery.

The primary end-points were to evaluate the safety of cytotoxic chemotherapy associated with hydralazine and magnesium valproate, as well as to evaluate changes in DNA methylation, HDAC inhibition and global gene expression before and after treatment. The secondary end-point was to evaluate clinical and pathological response after the fourth chemotherapy course. Clinical complete response (CR) response was defined as complete disappearance of all tumor signs in the breast and was assessed by both palpation and by the most appropriate imaging method. Partial response (PR) was defined as a reduction in the product of the two largest perpendicular diameters of primary-tumor size by 50% or more. In patients with multifocal or multicentric disease, solely the lesion with the largest diameter was assessed. Stable disease (SD) was defined as no significant increase or decrease in tumor size, and progressive disease was defined as the development of new, previously undetected lesions or ≥25% increase in the size of a pre-existing lesion. Pathological complete response (pCR) was defined as no microscopic evidence of residual viable tumor cells in breast and axillary lymph nodes. Toxicity during chemotherapy was evaluated according to common toxicity criteria (CTC) of National Cancer Institute (NCI) version 2.0.

### Clinical samples

Core-needle biopsies were taken from primary breast tumors at diagnosis and after 7 days of treatment with hydralazine and magnesium valproate prior to the first course of doxorubicin plus cyclophosphamide. Part of the biopsy was sent to the Institution's Pathology Department for routine hematoxilin and eosin diagnosis. The remaining biopsy specimen was immediately frozen at −20°C for biological analyses. In addition, a blood sample was drawn from the arm by venipuncture to obtain plasma and the mononuclear cell fraction.

### Nucleic acid extraction

Genomic DNA from tumors and peripheral mononuclear blood cells was obtained with the standard method of proteinase-K digestion and phenol-chloroform extraction. RNA from tumors was obtained using the TriReagent RNA extraction kit (Gibco BRL Grand Island, NY, USA) following manufacturer instructions.

### RT-PCR

Primers for *NDUFA13* were forward 5′aatgcaagaaccaaggcgagtcac3′and reverse 5′aggcatgtcctgcttcacctttga3, and for *DAPPER*, these were gcgaagagatgctggtttgt3′and 5′tgagagactcaaggtcgcc3′. Annealing temperatures were 60 and 55°C for 35 cycles. Products were electrophoresed and visualized under ultraviolet (UV) light.

### Global DNA methylation

Quantification of genomic 5-methylcytosine from peripheral blood DNA was performed by capillary electrophoresis, as previously described [27]. Relative methylation of each DNA sample was taken as the percentage of ^m^C in total cytosine: ^m^C peak area×100/(C peak area+^m^C peak area).

### Histone deacetylase assay

Assays were performed using the colorimetric HDAC activity assay from BioVision (BioVision Research Products, Mountain View, CA, USA) according to manufacturer instructions, as described [28].

### Valproic acid plasma levels

Valproic acid was measured in plasma using a fluorescence polarization immunoassay (FPIA) technology, as previously described [28].

### Hydralazine plasma levels

Hydralazine was determined in plasma by high-performance liquid chromatographic (HPLC) assay, as previously described [29].

### Microarray analysis

We used the Amersham (Piscataway, NJ, USA) CodeLink system containing 55,000 gene 30-mer oligonucleotide probes. Target preparation, microarray hybridization, post-hybridization processing and scanning, and normalization were done essentially as described [13]. Gene expression differences induced by epigenetic treatment were analyzed by comparing pre- and post-treated clinical samples versus normal breast serial analysis of gene expression (SAGE) libraries obtained from the SAGE public website. We included only libraries obtained from normal tissues and with a total amount of sequenced tags. Median expression values for each tag were calculated. To avoid errors in matching tag to Unigene cluster, we considered only tags of >30,000 for which a unique Unigene cluster match was found. To reduce redundancy due to different cluster ID-associated tags, we eliminated these repetitive Unigene IDs. Data values for each sample were normalized between the two platforms against the median of normal breast SAGE libraries, and Log2-based expression ratios were obtained. As unsupervised analysis, we performed hierarchical clustering and principal component analyses to group genes and samples on the basis of their similarities in expression as an initial analysis. An average linkage hierarchical-clustering technique was used in which the measurement of similarity was Spearman rank correlation among the samples' transcription profiles. Log-2 expression ratios were filtered to find genes with a 3-fold minimum expression ratio. Hierarchical clustering analyses and principal component analyses were performed by means of the Genesis program [30].

A supervised analysis was also performed to determine genes that were significantly differentially expressed between pre- and post-epigenetic treatment in clinical samples using significance analysis ofg microarrays. This method involves testing each gene one at a time and then performing 100 permutation tests to estimate the false- discovery rate that arises from input genes [31]. We selected the number of significant genes corresponding to a target false-discovery rate of 0.16.

### Statistical analysis

Comparisons between biological measurements before and after treatment were evaluated by Wilcoxon or unpaired *t* test. Analysis of the comparison between serum levels of hydralazine throughout treatment according to acetylator phenotype was carried out by independent Student *t* test. A *p* value <0.05 was considered significant.

## Results

### Patients

**Figure 1:**
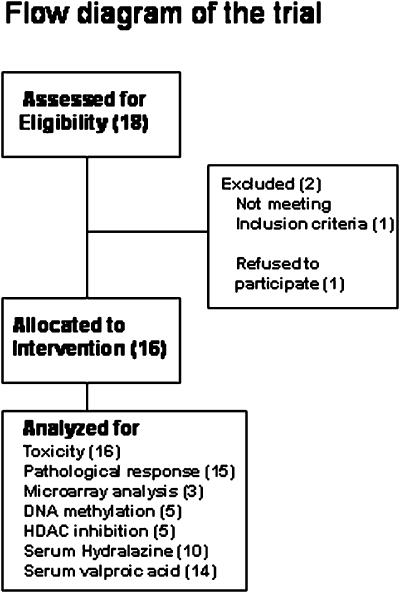
Flow diagram of the trial.

The flow diagram of the trial is shown in Figure 1. Sixteen patients were included in this study. Patient characteristics are listed in Table 1. Mean age was 47 years (range, 38–67 years), 68 and 32% were stages IIIA and IIB, respectively, and median tumor size was 5 cm (range, 3–12 cm). All patients had clinically positive nodes with median nodal size of 2 cm (range, 1–5 cm). The majority of tumors (80%) were ductal carcinomas, and 80% were intermediate- and high-grade tumors; 77% were estrogen receptor-positive and 30% were HER2- positive.

**Table 1:**
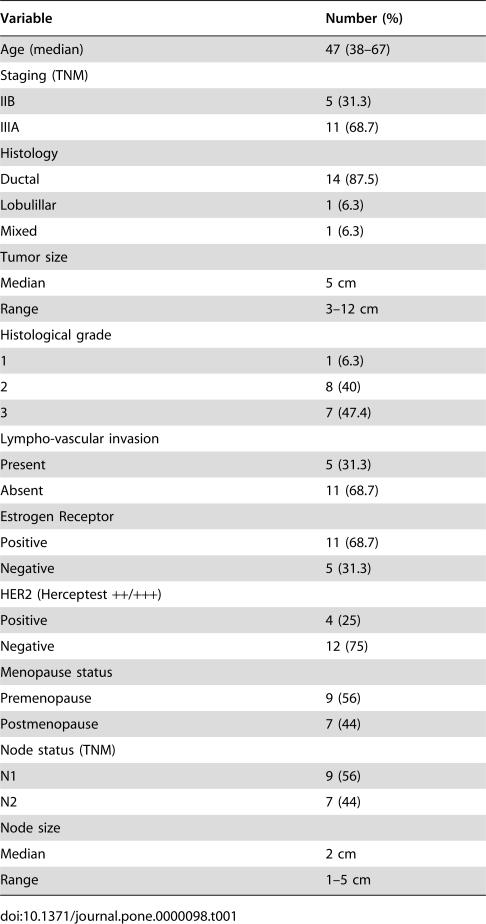
Clinicopathological characteristics

Variable	Number (%)
Age (median)	47 (38–67)
Staging (TNM)	
IIB	5 (31.3)
IIIA	11 (68.7)
Histology	
Ductal	14 (87.5)
Lobulillar	1 (6.3)
Mixed	1 (6.3)
Tumor size	
Median	5 cm
Range	3–12 cm
Histological grade	
1	1 (6.3)
2	8 (40)
3	7 (47.4)
Lympho-vascular invasion	
Present	5 (31.3)
Absent	11 (68.7)
Estrogen Receptor	
Positive	11 (68.7)
Negative	5 (31.3)
HER2 (Herceptest ++/+++)	
Positive	4 (25)
Negative	12 (75)
Menopause status	
Premenopause	9 (56)
Postmenopause	7 (44)
Node status (TNM)	
N1	9 (56)
N2	7 (44)
Node size	
Median	2 cm
Range	1–5 cm

All patients received four cycles of doxorubicin and cyclophosphamide plus hydralazine and magnesium valproate as planned. In addition, all patients were evaluated for clinical response and toxicity, but there were only 15 pathological assessments of the response, as one patient refused surgical treatment. Analysis of DNA methylation, HDAC activity before and after pre-treatment days with hydralazine and magnesium valproate was performed in five patients, whereas only three patients could be analyzed for global expression profiling of tumors. Hydralazine and valproic acid plasma levels were measured at several points during the protocol.

### Clinical Response

The sixteen patients were evaluable for clinical response. Five (31%) patients had complete response and eight (50%), partial response, for an overall response rate of 81%. Three patients had stable disease and none progressed (Table 2).

**Table 2:**
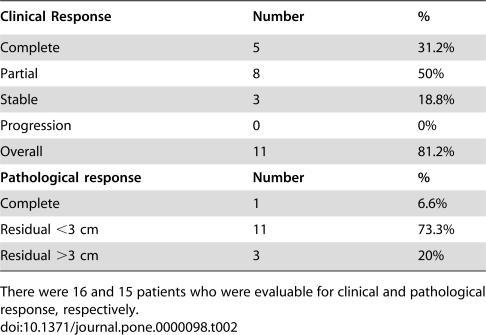
Response to treatment

Clinical Response	Number	%
Complete	5	31.2%
Partial	8	50%
Stable	3	18.8%
Progression	0	0%
Overall	11	81.2%
**Pathological response**	**Number**	**%**
Complete	1	6.6%
Residual <3 cm	11	73.3%
Residual >3 cm	3	20%

There were 16 and 15 patients who were evaluable for clinical and pathological response, respectively.

### Pathological Response

Fifteen patients underwent surgery. One patient (6.6%) had pathological complete response. However, in 70% of cases residual disease was <3 cm, 33% of cases had pathological negative lymph nodes, and no case had extranodal extension (Table 2).

### Toxicity

The treatment was well-tolerated in general. All patients received four cycles of pre-operative chemotherapy; in total, 64 cycles were administered. Hematological toxicity was the most common side effect, neutropenia grade 3/4 observed in 35% of cases and anemia grade 3/4 in 15% of cases. No episodes of grade 3/4 thrombocytopenia were registered. Only two patients presented febrile neutropenia. No toxic deaths were registered. Regarding non-hematological toxicity, drowsiness was the most frequent side-effect; it was observed in 31% of cycles, nonetheless in the vast majority, of grade 1 severity. Other side effects were tremor, edema, fatigue, nausea/vomiting, and headache, mainly grades 1 and 2 (Table 3).

**Table 3:**
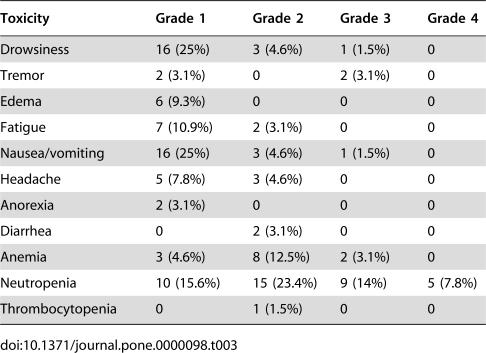
Toxicity of treatment from a total of 64 chemotherapy courses in the 16 patients

Toxicity	Grade 1	Grade 2	Grade 3	Grade 4
Drowsiness	16 (25%)	3 (4.6%)	1 (1.5%)	0
Tremor	2 (3.1%)	0	2 (3.1%)	0
Edema	6 (9.3%)	0	0	0
Fatigue	7 (10.9%)	2 (3.1%)	0	0
Nausea/vomiting	16 (25%)	3 (4.6%)	1 (1.5%)	0
Headache	5 (7.8%)	3 (4.6%)	0	0
Anorexia	2 (3.1%)	0	0	0
Diarrhea	0	2 (3.1%)	0	0
Anemia	3 (4.6%)	8 (12.5%)	2 (3.1%)	0
Neutropenia	10 (15.6%)	15 (23.4%)	9 (14%)	5 (7.8%)
Thrombocytopenia	0	1 (1.5%)	0	0

### DNA Methylation

**Figure 2:**
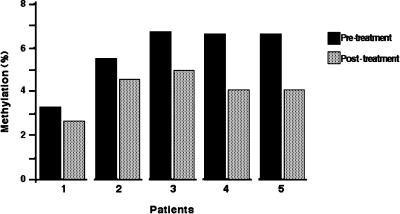
Global DNA methylation. The five patients had a reduction in 5mC content varying from 0.7−2.59% decrease. The mean 5mC in the five pre-treatment samples was 5.71% (standard deviation [SD], 1.43) which decreased to 4.2% (SD, 0.95). This difference was statistically significant (p = 0.043).

Global DNA methylation was assessed by capillary electrophoresis in DNA extracted from the peripheral blood of five patients. The results show in all cases a significant reduction in 5^m^C content, varying from 0.7–2.59% decrease. Mean 5^m^C in the five pre-treatment samples was 5.71% (standard deviation [SD], 1.43), which decreased to 4.2% (SD, 0.95). This difference was statistically significant (*p* = 0.043) (Figure 2).

### Histone Deacetylase Activity

**Figure 3:**
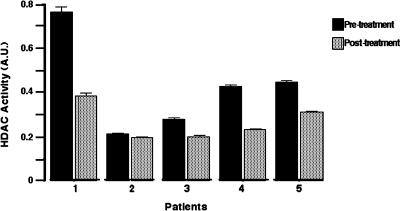
Histone deacetylase activity. The five patients assayed showed a reduction in enzymatic activity. In Y axis are the absolute values in optical density (OD) units. Higher and lower decreases were 0.3845 and 0.0175, for a mean decrease of 0.1624. This difference was statistically significant (p = 0.042).

HDAC enzymatic activity was tested in protein extracts from the peripheral blood of five patients. As shown in Figure 3, there was a reduction in enzymatic activity in these five patients as determined by optical density (OD)-unit absolute values. Higher and lower decreases were 0.3845 and 0.0175, for a mean decrease of 0.1624. This difference was statistically significant (*p* = 0.042).

### Valproic Acid Levels

Plasma samples for measuring valproic acid concentration throughout treatment were available for 14 patients. Mean concentrations varied from 78.5 µg/mL–100.3 µg/mL, for an overall mean of 87.5 µg/mL. There was a continuous decrease in mean concentrations from the beginning to the end of treatment; nevertheless, the sole statistically significant difference was observed when first and the last determinations were compared (*p* = 0.0118) (Table 4).

### Hydralazine Plasma Levels

Plasmatic levels of hydralazine were analyzed in 10 patients in whom plasma samples were available at different time-points during treatment. Distribution of these patients according to acetylator phenotype was four and six for slow and rapid, respectively. Mean plasma levels of hydralazine ranged from 204.8–275.1 ng/mL for rapid-acetylators, whereas these mean values were 252.1–344.2 ng/mL in slow- acetylators. Overall means were 246 and 299 ng/mL, respectively, which were not statistically significant different (*p* = 0.2445) (Table 5).

**Table 4:**
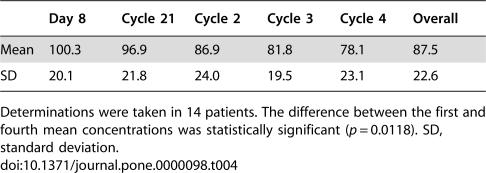
Plasmatic levels of valproic acid in µg/mL

	Day 8	Cycle 21	Cycle 2	Cycle 3	Cycle 4	Overall
Mean	100.3	96.9	86.9	81.8	78.1	87.5
SD	20.1	21.8	24.0	19.5	23.1	22.6

Determinations were taken in 14 patients. The difference between the first and fourth mean concentrations was statistically significant (*p* = 0.0118). SD, standard deviation.

**Table 5:**
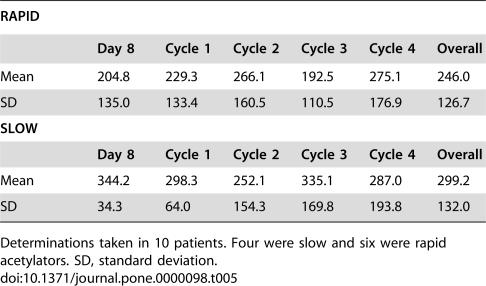
Hydralazine plasmatic levels according to the acetylator phenotype (ng/mL)

RAPID						
	Day 8	Cycle 1	Cycle 2	Cycle 3	Cycle 4	Overall
Mean	204.8	229.3	266.1	192.5	275.1	246.0
SD	135.0	133.4	160.5	110.5	176.9	126.7
**SLOW**						
	**Day 8**	**Cycle 1**	**Cycle 2**	**Cycle 3**	**Cycle 4**	**Overall**
Mean	344.2	298.3	252.1	335.1	287.0	299.2
SD	34.3	64.0	154.3	169.8	193.8	132.0

Determinations taken in 10 patients. Four were slow and six were rapid acetylators. SD, standard deviation.

### Gene Expression

**Figure 4:**
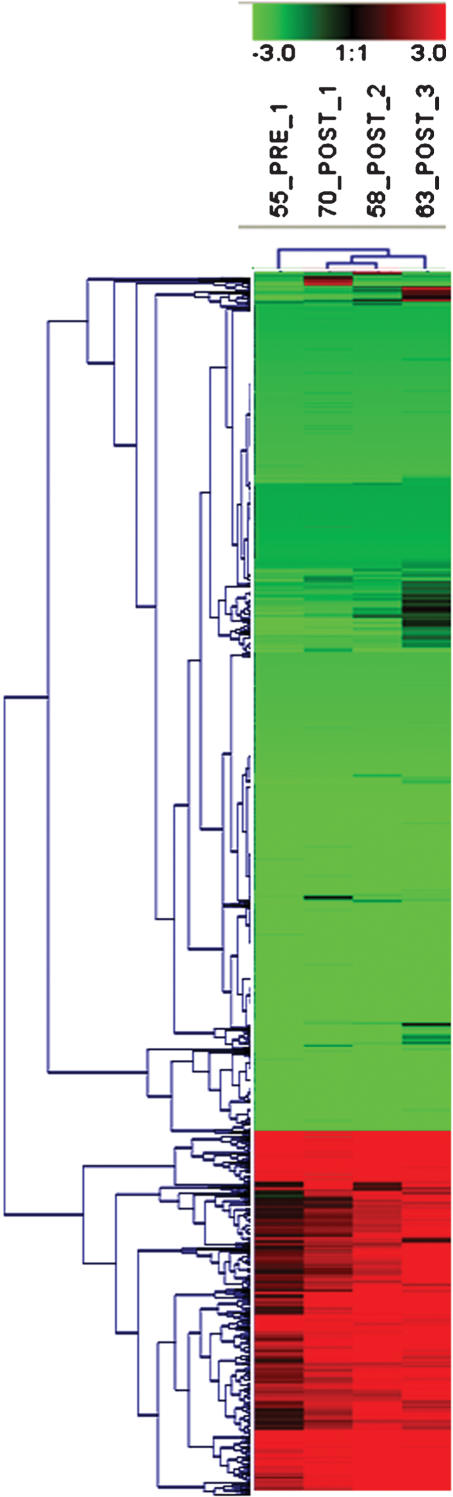
Hierarchical Cluster Analysis. The cluster shown represents 3,117 genes. Each row represents a gene, whereas each column corresponds to a tissue sample. The relative abundance of the gene in the tissue correlates with color intensity (red, induced; green, repressed; black, no change). On the dendogram, post-treated clinical samples clustered together.

**Figure 5:**
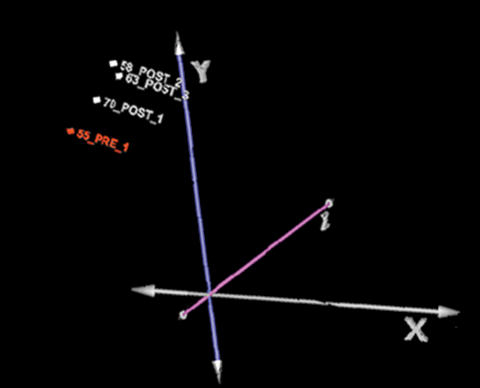
Principal Component Analysis. Principal Component Analysis, showing in three-dimensional space relationship between samples.

**Figure 6:**
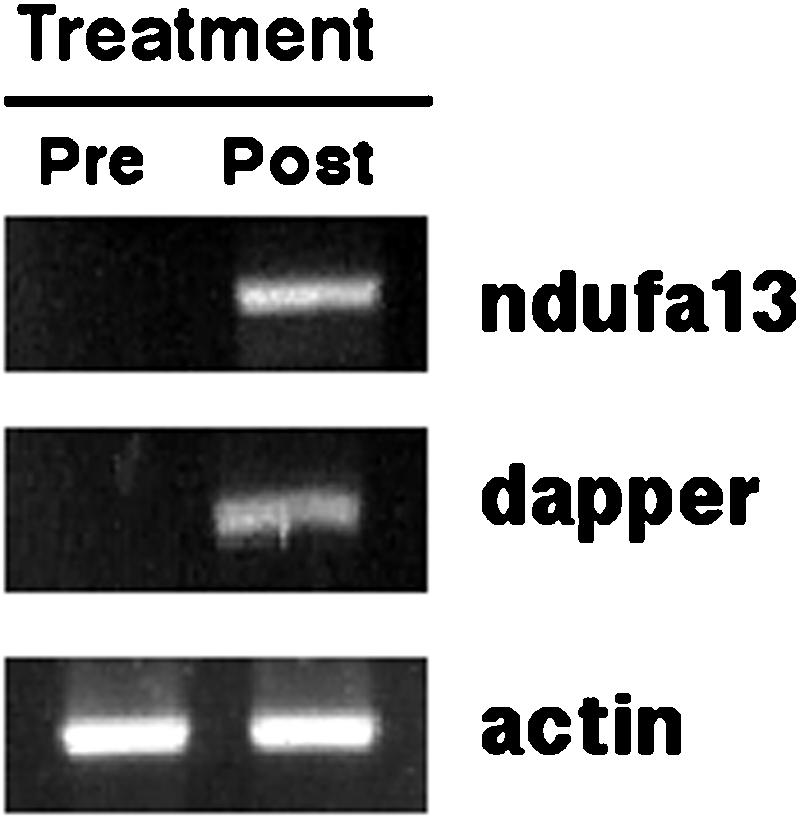
Gene expression by RT-PCR. RT-PCR of NDUFA13 and DAPPER genes in biopsies from the primary tumor showing that these genes were reactivated after treatment. This corresponds to one of the three patients whose biopsy (only post-treatment) was analyzed by microarray and showed over-expression of these genes.

A total of 3,117 genes were found up and down-regulated in clinical samples as compared with normal breast tissue. Figure 4 shows cluster analysis of microarray data. As can be observed, expression profile of post-treatment samples cluster together. This was also shown in a principal component analysis, which is an exploratory multivariate statistical technique that allows identification of variables in a multidimensional data set that explains differences or similarities between observations better than hierarchical clustering (Figure 5). With regard to changes in global gene expression observed after hydralazine and valproate treatment, the pre-treatment sample was compared against three post-treatment samples (GEO accession number GSE6304). The number of genes up- or down-regulated by an at least 3-fold difference was 1,091 and 89, respectively (Tables S1 and S2). Table 6 lists some selected genes found up- and down-regulated after treatment. To further assess the up-regulating effect of hydralazine and magnesium valproate, *NDUFA13* and *DRAPER* gene expression was assessed by RT-PCR in the single patient in whom RNA from pre- and post-treatment biopsies was available. As expected, expression of these genes was reactivated (Figure 6).

**Table 6:**
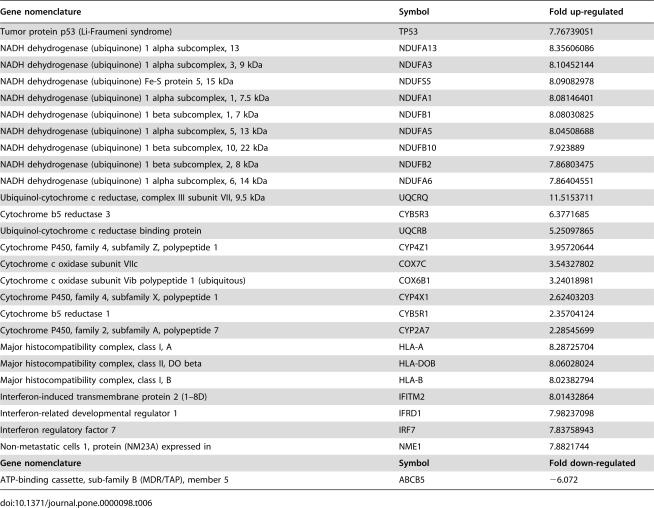
Selected up-regulated genes

Gene nomenclature	Symbol	Fold up-regulated
Tumor protein p53 (Li-Fraumeni syndrome)	TP53	7.76739051
NADH dehydrogenase (ubiquinone) 1 alpha subcomplex, 13	NDUFA13	8.35606086
NADH dehydrogenase (ubiquinone) 1 alpha subcomplex, 3, 9 kDa	NDUFA3	8.10452144
NADH dehydrogenase (ubiquinone) Fe-S protein 5, 15 kDa	NDUFS5	8.09082978
NADH dehydrogenase (ubiquinone) 1 alpha subcomplex, 1, 7.5 kDa	NDUFA1	8.08146401
NADH dehydrogenase (ubiquinone) 1 beta subcomplex, 1, 7 kDa	NDUFB1	8.08030825
NADH dehydrogenase (ubiquinone) 1 alpha subcomplex, 5, 13 kDa	NDUFA5	8.04508688
NADH dehydrogenase (ubiquinone) 1 beta subcomplex, 10, 22 kDa	NDUFB10	7.923889
NADH dehydrogenase (ubiquinone) 1 beta subcomplex, 2, 8 kDa	NDUFB2	7.86803475
NADH dehydrogenase (ubiquinone) 1 alpha subcomplex, 6, 14 kDa	NDUFA6	7.86404551
Ubiquinol-cytochrome c reductase, complex III subunit VII, 9.5 kDa	UQCRQ	11.5153711
Cytochrome b5 reductase 3	CYB5R3	6.3771685
Ubiquinol-cytochrome c reductase binding protein	UQCRB	5.25097865
Cytochrome P450, family 4, subfamily Z, polypeptide 1	CYP4Z1	3.95720644
Cytochrome c oxidase subunit VIIc	COX7C	3.54327802
Cytochrome c oxidase subunit Vib polypeptide 1 (ubiquitous)	COX6B1	3.24018981
Cytochrome P450, family 4, subfamily X, polypeptide 1	CYP4X1	2.62403203
Cytochrome b5 reductase 1	CYB5R1	2.35704124
Cytochrome P450, family 2, subfamily A, polypeptide 7	CYP2A7	2.28545699
Major histocompatibility complex, class I, A	HLA-A	8.28725704
Major histocompatibility complex, class II, DO beta	HLA-DOB	8.06028024
Major histocompatibility complex, class I, B	HLA-B	8.02382794
Interferon-induced transmembrane protein 2 (1–8D)	IFITM2	8.01432864
Interferon-related developmental regulator 1	IFRD1	7.98237098
Interferon regulatory factor 7	IRF7	7.83758943
Non-metastatic cells 1, protein (NM23A) expressed in	NME1	7.8821744
**Gene nomenclature**	**Symbol**	**Fold down-regulated**
ATP-binding cassette, sub-family B (MDR/TAP), member 5	ABCB5	−6.072

## Discussion

The idea of treating cancer patients with agents able to re-establish expression of tumor suppressor genes silenced by epigenetic mechanisms is currently being tested. Clinical trials using a combination of DNA methylation and HDAC inhibitors have already shown promising results in hematological neoplasms [9], [10]. Here we report on the safety and the biological and clinical efficacy of hydralazine and magnesium valproate added to doxorubicin cyclophosphamide for neoadjuvant treatment of locally advanced breast cancer.

The results of this proof-of-principle study demonstrate that this therapy is safe, that it achieves the molecular changes expected from use of a demethylating and an HDAC inhibitor and appear to increase the efficacy of conventional cytotoxic agents.

Patients entering the study had IIB- and IIIA-stage disease with median tumor size and median axillary nodal sizes of 5 and 2 cm, respectively. Despite this, we observed clinical complete response in five (31.2%) patients, for an overall response rate of 81%. This rate is in the upper limit of response rates observed across multiple trials according to a recent meta-analysis that reports overall responses between 45 and 83% [32]. Regarding the pathological response, only one of 15 patients (6.6%) achieved complete response. This response rate is, nonetheless, within the range expected from four cycles of anthracyclin-based chemotherapy with rates varying between 3 and 16% [23], [25], [33]–[38]. Notwithstanding this, we have no historical control at our Institution treated with this regimen to better assess whether addition of hydralazine and magnesium valproate increased the response. Nevertheless, a recent study in patients with breast cancer treated with anthracyclin-based neoadjuvant chemotherapy at a Mexican institution reports 7% and 45% complete and partial responses, respectively, none pathological complete [39]. Regarding the response in axillary nodes at treatment initiation, all patients had clinically positive nodes, while after therapy we found only 10 patients with pathologically axillary nodes involved. In this regard, Rouzier et al. recently reported on 152 patients with T1–3 breast cancer and positive axillary nodes. After neoadjuvant chemotherapy, 23% had complete disease eradication in axillary lymph nodes [40]. In the present study, axillary pathological complete response rate was 33%; however, no cytological confirmation of pre-treatment status was performed in our study.

Recently, a nomogram based on clinical stage, estrogen receptor status, histological grade, and number of pre-operative chemotherapy cycles that had good discrimination and calibration in training and anthracyclin-treated validation sets has become available to the public [41]. To obtain further information on the usefulness of our treatment protocol, data of the 15 patients were entered into the nomogram, and we found that predicted pathological complete response rate for our patients was 12%, whereas the actual rate observed was 6.6%. Nonetheless, the probability of achieving a pathological residual <3 cm as predicted from the nomogram was 39%, but the observed rate was 73.3%. These results suggest that the addition of hydralazine and magnesium valproate to the four doxorubicin-cyclophosphamide cycles may have increased probability of response.

Regarding the safety of cytotoxic chemotherapy-associated hydralazine and magnesium valproate, this treatment was well-tolerated. The only toxicity that could be attributed to the experimental therapy―specifically, to valproate―was drowsiness in the majority of patients; however, this was grades 1 and 2, which by definition do not interfere with patient functioning in daily living. Only one patient presented grade-3 drowsiness. Regarding hydralazine, we observed headache and leg edema but no flushing, hypotension, palpitation, tachycardia, dizziness, or angina pectoris, which comprise hydralazine's cardiovascular effects. The lack of minor side effects produced by these drugs can be at least partly due to the slow-release formulation that avoids hydralazine plasma peaks. Hematological toxicity was within ranges reported by anthracyclin-based schemas, with no grade 3/4 thrombocytopenia, and 15% grade 3/4 anemia; nevertheless, we observed grade 3/4 neutropenia in 35% of cases, which appears to be higher than that reported by other authors [23], [25], [33]–[38]. Valproic acid is known to induce trilineage hematological toxicity [42] and to increase the hematological toxicity of a regimen containing fotemustine and cisplatin [43]; nonetheless, it has also been reported that this drug stimulates hematopoietic stem cell proliferation and self-renewal [44]. Whether or not the combination of hydralazine and valproate potentiated chemotherapy-induced myelosuppression needs to be further evaluated.

Regarding plasmatic levels achieved with the treatment, it is remarkable that the hydralazine levels achieved were nearly 3-fold higher than those commonly observed when hydralazine is used as an antihypertensive [45]. In our previous phase I study using hydralazine at doses between 50 and 150 mg/day without accounting for acetylator phenotype, we observed demethylating and reactivating effects [46]; hence, the levels observed herein readily explain the molecular changes observed regarding methylation and gene expression. In this regard, pharmacokinetic characterization in healthy volunteers of the slow-release hydralazine formulation employed in this study (unpublished data) demonstrated AUC concentrations of 6,034+1,899 ng/h/mL and 2,751+954 ng/h/mL for slow- and rapid-acetylators, respectively, using 182 mg regardless of acetylator status. It is noteworthy that the dose adjustment we chose of 182 mg/day and 83 mg/day for rapid-and slow-acetylators, respectively, was adequate, because no statistically significant differences were found in hydralazine plasmatic levels between both types of patients (means, 246 and 249 ng/mL,respectively). With respect to valproic acid levels, we found a mean concentration of 87.5 µg/mL, which is within ranges observed in our previous phase I study demonstrating histone hyperacetylation and HDAC inhibition, in which mean concentrations observed were 94.06 µg/mL, 123.46 µg/mL, and 90.93 µg/mL for dose levels of 20, 30, and 40 mg/kg, respectively. Plasma levels progressively decreased until the end of treatment. However, the mean value in the lowest (four-cycle) determination was >75 µg/mL, which is an effective concentration [28].

There is a very large number of *in vitro* studies demonstrating global and gene-promoter DNA hypomethylation after treatment with nucleoside analogs, principally 5-azacytidine and 5-aza-2-deoxyazacytidine [46]. Interestingly, this hypomethylating effect has also been achieved in a number of clinical trials employing these agents in hematological malignancies; in fact, measurement of this biological effect constitutes a surrogate marker for the activity of these agents [47]. The same has been observed regarding H3 and H4 histone hyperacetylation and/or inhibition of HDAC activity by a number of agents including valproic acid [48]. Here, we confirmed our *in vitro* [49], [50] and clinical observations [51] that hydralazine is an effective DNA methylation inhibitor that led to a statistically significant reduction in the percentage of 5^m^C content in peripheral mononuclear cells after 7 days of treatment. Interestingly, the hypomethylating effect appears higher, or at least similar, to that achieved by 5-aza-2-deoxyazacytidine when evaluated by a related quantitative HPLC-based assay after 7 days of continuous infusion [52]. Other authors have also demonstrated the ability of hydralazine to hypomethylate DNA in other experimental systems [2006, ASCO Procc Abstr 13131], as well as in a clinical setting. Zafar et al. have shown that hydralazine used together with neoadjuvant chemotherapy decreases DNA methylation in primary tumors of patients with breast cancer [2006, AACR Procc 2006 Abstr 41].

On the other hand, the ability of valproic acid to hyperacetylate H3 and H4 histones and to inhibit HDAC has been also demonstrated in the peripheral blood of patients with hematological neoplasms [10], [9]. Despite this, it is not totally clear whether histone hyperacetylation is the sole mechanism for explaining its antitumor effects; in vitro studies have demonstrated a good correlation between these phenomena [54]. Our findings of a significant decrease in histone acetylase activity as measured by an ELISA assay in five of five patients further support the clinical use of this agent.

A number of pre-clinical studies have consistently demonstrated that DNA demethylating agents and HDAC inhibitors, either alone or in combination, are able to modulate the expression of a number of genes in ranges varying from 0.5–12% [55], [56], and that both classes of agents synergize not only the antitumor effects [4]–[8], but also the extent of gene up-regulation [2], [3]. We recently confirmed this synergy between hydralazine and valproic acid regarding global gene expression by studying colon carcinoma cell line SW480, in which hydralazine and valproic acid led to up-regulation of 153 and 178 genes, respectively, whereas the number of up-regulated genes increased to 352 when used in combination [13]. These data led us to evaluate gene-expression changes at a global level by use of the CodeLink microarray system after 7 days of treatment with this combination of epigenetic agents. Despite that we could only evaluate three patients, among whom only one yielded sufficient RNA in the pre-treatment sample, our data clearly demonstrated the ability of these agents to up-regulate 1,091 genes and to down-regulate 89. As this is the first study evaluating the effect of epigenetic therapy in solid tumors with this methodology, we have no means of comparison with similar studies; nonetheless, it is remarkable that the extent of up-regulation falls well within that expected from in vitro experiments of global expression, including that performed by our group with the same method of analysis [13]. It should also be noted that in two microarray studies performed in primary breast-tumor samples before and after 24–72 h after chemotherapy, the up-regulated number of genes is ca 20% of those observed in our study [57], [58]. These data, along with our findings of significant DNA hypomethylation and HDAC inhibition, strongly indicate that hydralazine and magnesium valproate up-regulate the expression of a significant number of genes in a clinical scenario. Another supportive finding on their reactivating activity was the unexpected observation that the three post-treatment samples cluster together (Figures 4 and 5) when the four samples were compared with gene expression of normal breast.

Regarding the nature of gene-expression changes, Table 6 list selected genes that showed expression changes (the full list is supplied as Supporting Information). The data are quite interesting and we emphasize the effect observed in tumor suppressor gene p53. Hydralazine and magnesium valproate induced 7.7-fold p53 expression, which confirms that HDACs can up-regulate p53 gene transcription, as demonstrated with depsipeptide and trichostatin A [59], [60]. Moreover, it has been shown that p53 induction by HDAC inhibitors may completely deplete mutant p53, and such a sudden restoration is highly cytotoxic to cells harboring mutant p53 [59]. On the other hand, the induction of functional p53 induced by depsipeptide sensitizes SW-1736 cells to doxorubicin [60]. The modulation of this gene for therapeutic aims can be better highlighted from studies using short peptides to rescue its function [61], as well as their local delivery into tumors by viral vectors, as in a recent study that demonstrated its potential to increase chemotherapy effects in patients with breast cancer [62]. On the other hand, we observed over-expression of IFN-response pathway genes, which have been found consistently up-regulated by epigenetic drugs in a number of experimental systems and which increase recombinant interferon-alpha cytotoxicity [63]. Likewise, epigenetic agents have also been shown to up-regulate class-I molecules of the major human histocompatibility complex [64]. Here we show that hydralazine and magnesium valproate led to a >8-fold up-regulation in class-I A and -B molecules. This may also have sound therapeutical implications in the treatment of cancer. A well-known mechanism that tumors utilize to evade antitumor host-response comprises the transcriptional silencing of molecules of the tumor recognition complex, such as class-I molecules [64]. In fact, a recent clinical study on renal cancer and melanoma utilizing a DNA methylation inhibitor along with high-dose interleukin-2 achieved encouraging response rates attributed to facilitation of the immune response against the tumor [65]. In this regard, we have gathered data (submitted for publication) that hydralazine and valproate not only up-regulate HLA-class-I molecules, but also tumor-associated MAGE- and GAGE-family antigens in cervical cancer-cell lines, which increases the immune recognition and killing of these cervical cancer-cell lines by HPV peptide-stimulated autologous T cells. Additionally of note is up-regulation of the antimetastatic gene product NM23, which is frequently methylated and silenced in patients with breast cancer [66].

At least―but not in the least―we were surprised by large number of up-regulated genes belonging to the mitochondrial oxidative phosphorylation (OXPHOS) system, with a total of 18 members, the majority of these from complex I. The OXPHOS system consists of five multiprotein complexes, the individual subunits of which are encoded either by the mitochondrial or by the nuclear genome. Defects in the OXPHOS system result in devastating diseases, and in recent years its role in cancer has begun to be studied [67]. For example, the NADH dehydrogenase (ubiquinone) 1 alpha subcomplex 13 is a gene known to be down-regulated in basal cell carcinomas [68]. This gene, also denominated *NDUFA13* or *GRIM19*, was initially identified as a pro-apoptotic gene mediating retinoid antitumor effects and indispensable for proper assembling and functioning of complex I of this pathway [69]. In addition, it has been demonstrated that NADH dehydrogenase plays an important role in generating ROS during doxorubicin treatment, and its down-regulation has been found in doxorubicin-resistant A431 cells [70]. This was one of the two individual genes we assessed in the pre-and post-treatment biopsies by RT-PCR in a single patient corroborating the up-regulation observed in the microarray analysis.

Regarding down-regulated genes, in this work we solely point out that ATP-binding cassette, sub-family B (MDR/TAP), member 5 was down-regulated by hydralazine and magnesium valproate treatment. Interestingly, ABCB5 is a novel drug transporter and a chemoresistance mediator identified in human melanoma and instinctively present in a subset of chemoresistant, stem cell phenotype-expressing tumor cells among melanoma bulk populations, indicating that these chemoresistant cells can be specifically targeted via ABCB5 to enhance cytotoxic efficacy [69].

### Conclusion

In this proof-of-concept study, we demonstrate that treatment with hydralazine and magnesium valproate exerts its proposed molecular effects of DNA demethylation, HDAC inhibition, and gene reactivation in primary tumors of patients with breast cancer. Importantly, this doxorubicin- and cyclophosphamide-associated treatment is safe and well-tolerated, and appears to increase the efficacy of chemotherapy. A randomized phase III study is ongoing to support the efficacy of the so-called epigenetic or transcriptional cancer therapy.

## Supporting Information

Table S1 Up-regulated genes
(0.17 MB XLS)

Table S2 Down-regulated genes
(0.03 MB XLS)
